# The Ambiguous Relationship of Oxidative Stress, Tau Hyperphosphorylation, and Autophagy Dysfunction in Alzheimer's Disease

**DOI:** 10.1155/2015/352723

**Published:** 2015-06-15

**Authors:** Zhenzhen Liu, Tao Li, Ping Li, Nannan Wei, Zhiquan Zhao, Huimin Liang, Xinying Ji, Wenwu Chen, Mengzhou Xue, Jianshe Wei

**Affiliations:** ^1^Institute of Neuroscience, Henan Polytechnic University, Jiaozuo 454000, China; ^2^Laboratory of Brain Function and Molecular Neurodegeneration, Institute for Brain Science Research, School of Life Sciences, Henan University, Kaifeng 475004, China; ^3^Department of Neurology, The First Affiliated Hospital, Henan University, Kaifeng 475000, China; ^4^Institute of Neurological Disorder, Henan University, Kaifeng 475000, China

## Abstract

Alzheimer's disease (AD) is the most common form of dementia. The pathological hallmarks of AD are amyloid plaques [aggregates of amyloid-beta (A*β*)] and neurofibrillary tangles (aggregates of tau). Growing evidence suggests that tau accumulation is pathologically more relevant to the development of neurodegeneration and cognitive decline in AD patients than A*β* plaques. Oxidative stress is a prominent early event in the pathogenesis of AD and is therefore believed to contribute to tau hyperphosphorylation. Several studies have shown that the autophagic pathway in neurons is important under physiological and pathological conditions. Therefore, this pathway plays a crucial role for the degradation of endogenous soluble tau. However, the relationship between oxidative stress, tau protein hyperphosphorylation, autophagy dysregulation, and neuronal cell death in AD remains unclear. Here, we review the latest progress in AD, with a special emphasis on oxidative stress, tau hyperphosphorylation, and autophagy. We also discuss the relationship of these three factors in AD.

## 1. Introduction

Alzheimer's disease (AD) is the most common form of dementia in the elderly and a chronic neurodegenerative disease characterized by widespread degeneration of neurons. An estimated 37 million people worldwide currently have AD, which is estimated to increase to 65.7 million by 2030 and 115.4 million by 2050 [1, 2]. AD is a growing health concern in society because patients suffer from progressive functional impairments, emotional distress, loss of independence, and behavioral deficits. It is characterized by the presence of two types of neuropathological hallmarks: senile plaques (SPs) and intracellular neurofibrillary tangles (NFTs). SPs predominantly consist of extracellular amyloid *β*-peptide (A*β*) deposits. NFTs are formed by intraneuronal aggregation of hyperphosphorylated tau. The amyloid cascade hypothesis theory proposes a dysregulation of amyloid precursor protein processing. This event leads to AD pathogenesis, which involves the aggregation of A*β* (particularly A*β*42), neuritic plaque formation, and consequently the formation of NFTs followed by the disruption of synaptic connections, neuronal death, and cognitive deficits (dementia) [3]. Increasing evidence suggests that A*β* oligomers (A*β*Os) may be the primary cause of AD because they have a greater correlation with dementia than insoluble A*β*42 [4]. These A*β*Os bind to a putative receptor and activate the receptor tyrosine kinase EphA4 and Fyn. A*β*Os binding triggers aberrant activation of NMDARs and abnormal increase in postsynaptic Ca^2+^. The following events include increased generation of reactive oxygen species (ROS), and membrane lipid peroxidation; mitochondrial fragmentation, Ca^2+^ induced Ca^2+^ release (CICR), then produces altered surface expression and dysregulation of receptor function, excitotoxicity, dendritic spine retraction, and elimination [4–6].

A*β* also plays a crucial role in inducing neuronal oxidative stress [7, 8]. A*β*-mediated mitochondrial oxidative stress causes hyperphosphorylation of tau in AD brains [8–10]. Mounting evidence clearly links tau to neurodegeneration, indicating that tau hyperphosphorylation may be the necessary point in neural dysfunction and death. However, whether autophagic dysfunction is involved in neuronal death during this event still remains unknown. Recent studies have indicated the importance of defective autophagy in the pathogenesis of aging and neurodegenerative diseases [11–14], especially in AD [15–17]. Autophagy may increase the formation of autophagosome in AD, and autophagic dysfunction may induce the pathogenesis of AD, particularly at the late stage of AD [18–22]. However, the relationship between oxidative stress, tau protein hyperphosphorylation, autophagic dysfunction, and neuronal cells death in AD remains elusive. In this review, we summarize the latest progress in research focused on oxidative stress, tau hyperphosphorylation, and autophagic dysfunction and their relationship with AD.

## 2. Oxidative Stress in AD

In experimental models and human brain studies of AD, oxidative stress has been shown to play an important role in neurodegeneration [10, 23, 24]. Generally, oxidative stress is caused by the imbalance between reactive oxygen species (ROS) (O_2_^−^, H_2_O_2_, HO_2_, and ·OH) and the breakdown of chemically reactive species, by reducing agents and antioxidant enzymes, such as manganese superoxide dismutase (SOD_2_) [25, 26]. This disequilibrium may result from disease, stressors, or environmental factors. High ROS levels lead to the accumulation of oxidized proteins, lipids, and nucleic acids, thereby directly impairing cellular function if not removed or neutralized [27]. Oxidative damage to cellular components is likely to result in the alteration of membrane properties, such as fluidity, ion transport, enzyme activities, protein cross-linking, and eventually cell death.

Oxidative stress has been reported to be one of the earliest events in AD. Several risk factors for AD may cause or promote oxidative damage, such as advanced age [28, 29] and apolipoprotein E (APOE) *ε*4 alleles [30, 31]. Medical risk factors include traumatic brain injury [32], stroke [33], hypertension [34], diabetes mellitus [35], hypercholesterolemia [36], and hyperhomocysteinemia [37]. Environmental and lifestyle-related risk factors include aluminum exposure [38], smoking [39], high calorie intake [40], vitamin D deficiency [41], lack of exercise [42], and lack of intellectual activities [43–45]. Mitochondrial dysfunction is known to be associated with oxidative stress and thus may be an initial trigger for enhanced A*β* production during the aging process [46–48]. Both soluble and fibrillar A*β* may further accelerate oxidative stress, as well as mitochondrial dysfunction [49, 50]. The transgenic (Tg) Thy1-APP751 (SL) mouse model of AD shows increased proteolytic cleavage of APP, increased production of A*β*, and impaired Cu/Zn-SOD activity [51]. Furthermore, oxidative stress is considered as a primary factor of NFT formation in AD [10, 24, 52, 53]. However, the relationship between oxidative stress and tau hyperphosphorylation remains unclear. Okadaic acid is used as a research model to induce tau phosphorylation and neuronal death in AD. Oxidative stress combined with okadaic acid results in tau hyperphosphorylation [54]. Mitochondrial SOD_2_ deficiency increases the levels of Ser396 phosphorylated tau in the Tg2576 mouse model of AD [55].

## 3. Tau Protein in AD

### 3.1. Tau Protein Physiology and Pathology

Tau protein (known as neuronal microtubule associated protein tau) plays a large role in the outgrowth of neuronal processes and the development of neuronal polarity [56–58]. Tau protein in the central nervous system is predominantly expressed in neurons [59, 60], with its main function to promote microtubule assembly, stabilize microtubules, affect the dynamics of microtubules in neurons [61, 62], and inhibit apoptosis [63], particularly in axons [64, 65]. However, recent reports suggest that excess intracellular tau is released into the extracellular culture medium via membrane vesicles [66]. In the adult human brain, tau consists of six isoforms, and the tau gene contains 16 exons. These isoforms are generated by alternative splicing of exons 2, 3, and 10 of its pre-mRNA [67, 68]. The six tau isoforms differ from each other by the presence or absence of one or two inserts (coded by exon 2 or exons 2 and 3) in the N-terminal part and the presence or absence of the second microtubule-binding repeat (encoded by exon 10) in the C-terminal portion. Depending on the alternative splicing of exon 10, tau isoforms are termed 4R (four microtubule-binding domains, with exon 10) or 3R (three microtubule-binding domains, without exon 10). Adult human brain expresses both 3R-tau and 4R-tau, whereas fetal human brain expresses only 3R-tau [69, 70]. Immunocytochemistry and biochemical analysis indicate that the ratio of 3R- to 4R-tau altered in AD and other neurodegenerative brain disorders [71–73], although in the normal adult human brain the level of 3R-tau is approximately equal to that of 4R-tau [74].

Tau protein normally stabilizes axonal microtubules in the cytoskeleton and plays a vital role in regulating the morphology of neurons. It has more than 30 phosphorylation sites. When tau is abnormally hyperphosphorylated, it destabilizes microtubules by decreasing the binding affinity of tau, affecting its axonal transport and resulting in its aggregation in NFTs [64]. NFTs are composed of paired helical filaments (PHF) of abnormally hyperphosphorylated tau. The pathogenesis of tau-mediated neurodegeneration is unclear but hyperphosphorylation, oligomerization, fibrillization, and propagation of tau pathology have been proposed as the likely pathological processes that induce the loss of function or gain of tau toxicity, which caused neurodegeneration [75]. Tau phosphorylation has been investigated at AD-related sites by using recombinant human tau phosphorylated by DNA damage-activated checkpoint kinase 1 (Chk1) and checkpoint kinase 2 (Chk2) in vitro [76]. This study identified a total of 27 Ser/Thr residues as Chk1 or Chk2 target sites. Among these sites, 13 have been identified to be phosphorylated in AD brains [77]. The generation of a Tg mouse line overexpressing human tau441 via V337M and R406W tau mutations has been shown to accelerate the phosphorylation of human tau, inducing tau pathology and cognitive deficits [78]. Pseudophosphorylation of tau reduces microtubule interactions, disrupts the microtubule network, and exerts neurotoxicity [79]. Interestingly, doubly pseudophosphorylated tau proteins enhance microtubule assembly activity and are more potent at regulating dynamic instability [80]. However, four singly pseudophosphorylated tau proteins exhibit a loss of function at the same sites (Thr [231], Ser [262], Ser [396], and Ser [404]) [80].

### 3.2. Tau Protein Kinases and Phosphatase

Tau phosphorylation is mainly determined by a balance between the activation of various tau protein kinases and phosphatases and its disruption results in the abnormal phosphorylation of tau, which is observed in AD. Each tau site is phosphorylated by one or more protein kinases. Tau kinases are grouped into three classes: (1) proline-directed protein kinases (PDPK) containing glycogen synthase kinase-3 (GSK3), dual specificity tyrosine-phosphorylation-regulated kinase 1A/B (Dyrk1A/B), cyclin-dependent protein kinase-5 (CDK5), and mitogen activated protein kinases (MAPK) (e.g., p38, Erk1/2, and JNK1/2/3); (2) non-PDPK, including tau-tubulin kinase 1/2 (e.g., casein kinase 1*α*/1*δ*/1*ε*/2), microtubule affinity regulating kinases, phosphorylase kinase, cAMP-dependent protein kinase A (PKA), PKB/AKT, protein kinase C, protein kinase N, and Ca^2+^/calmodulin-dependent protein kinase II (CaM kinase II); and (3) tyrosine protein kinases, including Src family kinase (SFK) members (e.g., Src, Lck, Syk, and Fyn) and Abelson family kinase members, ABL1 and ABL2 (ARG).

GSK3 (particularly GSK3*β*) plays a key role in the pathogenesis of AD, contributing to A*β* production and A*β*-mediated neuronal death by phosphorylating tau in most serine and threonine residues and inducing hyperphosphorylation in paired helical filaments [81]. Inhibition of GSK3 prevents A*β* aggregation and tau hyperphosphorylation [82, 83]. The involvement of CDK5 in tau phosphorylation is shown by the increase in its enzymatic activity and the absence of MT-2 cells neurite retraction in the presence of roscovitine or CDK5 siRNA [84]. Therefore, CDK5 may be a key candidate target for therapeutic gene silencing [85]. p38 MAPK has been identified as one of the kinases involved in the regulation of tau phosphorylation. Thus, under pathological conditions this kinase is likely to play a role in the hyperphosphorylation of tau [86]. CDKs and casein kinase 1 (CK1) are involved in the aggregation of A*β* peptides (forming extracellular plaques) and hyperphosphorylation of tau (forming intracellular NFTs). The expression pattern of CKI*δ* (an isoform of CK1) plays an important role in tau aggregation in AD [87]. Ser214, Ser262, and Ser409 are major phosphorylation sites of tau that are affected by PKA [88]. In P19 cells stably expressing human tau441, CaM kinase II has been shown to be involved in retinoic acid- (RA-) induced tau phosphorylation-mediated apoptosis [89].

Phosphatases are also usually classified into three classes according to their amino acids sequences, the structure of their catalytic site, and their sensitivity to inhibitors. These groups include (1) phosphoprotein phosphatase (PPP), (2) metal-dependent protein phosphatase, and (3) protein tyrosine phosphatase (PTP). Tau phosphatases belong to the PPP group (protein phosphatase [PP] 1, PP2A, PP2B, and PP5) and PTP group tumor suppressor phosphatase and tensin homolog (PTEN). The activity of PP2A, PP1, PP5, and PP2B accounts for approximately 71%, 11%, 10%, and 7%, respectively, in the normal human brain. However, in the AD brain, the total phosphatase activity (and including overall activity) for tau of PP2A, PP1, and PP5 is significantly decreased by 50%, 20%, and 20%, respectively [90]. PP2A contributes to abnormally hyperphosphorylated tau protein and is the most efficient phosphatase. Moreover, the inhibition of PP2A significantly plays a role in tau hyperphosphorylation [91–93]. It indicated PP2A is downregulated in the Down syndrome (DS) brain and thus may be involved in the abnormal hyperphosphorylation and accumulation of tau [94].

PP2A is regulated by endogenous inhibitor-1 of PP2A (I1PP2A) and inhibitor-2 of PP2A (I2PP2A) in mammalian tissues [95]. In AD brain, I2PP2A is translocated from neuronal nucleus to cytoplasm where it inhibits PP2A activity and promotes abnormal phosphorylation of tau. With inactivation of the nuclear localization signal (NLS) of I2PP2A, ^179^KRK^181^ → ^179^AAA^181^ along with ^168^KR^169^ → ^168^AA^169^ mutations in I2PP2A (mNLS-I2PP2A), I2PP2A was translocated from nucleus to the cytoplasm. Cytoplasmic retention of I2PP2A physically interacted with PP2A and inhibited its activity and induced Alzheimer-like abnormal tau protein hyperphosphorylation by the direct interaction of I2PP2A with PP2A and GSK-3*β* [96]. I2PP2A directly inhibits the activity of PP2A without affecting its expression [97]. GSK-3 activation significantly contributes to tau hyperphosphorylation by inhibiting PP2A via the upregulation of I2PP2A [98]. Okadaic acid is also considered to be a selective and potent inhibitor of serine/threonine phosphatase-1 and PP2A, which induces hyperphosphorylation of tau under in vitro and in vivo conditions [99]. These data indicate that upregulation or downregulation of the phosphorylation system or dephosphorylation system, respectively, of tau protein may be implicated in tau pathologies.

### 3.3. Tau Protein and Oxidative Stress

#### 3.3.1. Tau Protein Hyperphosphorylation and Oxidative Stress

Oxidative stress is believed to be a prominent early event in the pathogenesis of AD, contributing to tau phosphorylation and the formation of neurofibrillary tangles [48]. However, the relationship and underlying mechanisms between oxidative stress and tau hyperphosphorylation remain elusive. Fatty acid oxidative products provide a direct link between the mechanisms of how oxidative stress induces the formation of NFTs in AD [100]. Data from experiments show that chronic oxidative stress increases the levels of tau phosphorylation at paired helical filaments (PHF-1) epitope (serine 396/404) via the inhibition of glutathione synthesis with buthionine sulfoximine (BSO) in an vitro model of chronic oxidative stress [9]. In primary rat cortical neuronal cultures stimulated by the combination of the copper chelator, cuprizone, and oxidative stress (Fe^2+^/H_2_O_2_), tau phosphorylation is significantly increased by the elevated activity of GSK-3 [101]. Furthermore, treatment of rat hippocampal cells and SHSY5Y human neuroblastoma cells with H_2_O_2_ at the early stages of oxidative stress exposure results in tau dephosphorylation at the tau1 epitope by CDK5 via PP1 activation [102]. Several studies have suggested that oxidative stress is a causal factor in tau-induced neurodegeneration in* Drosophila* [103–105]. In contrast, a fragment of tau protein has been shown to induce copper reduction, thus contributing to oxidative stress and initiating copper-mediated generation of H_2_O_2_ [106].

#### 3.3.2. GSK3*β*, PP2A, and Oxidative Stress

Oxidative stress is likely to play a critical role in tau hyperphosphorylation, which is regulated by tau protein kinase activation and the suppression of phosphatase. Tau hyperphosphorylation may be induced by oxidative stress through the direct interaction with tau protein kinase and phosphatase, particularly GSK-3*β* and PP2A, respectively, because they are predominant and play an important role. A recent study has indicated that GSK-3*β* activity is upregulated under oxidative stress [107]. In human embryonic kidney 293/tau cells, H_2_O_2_ increases GSK-3*β* activity and tau is hyperphosphorylated at Ser396, Ser404, and Thr231 [107]. Mitochondrial superoxide activates the mitochondrial fraction of GSK-3*α*/*β*, resulting in the phosphorylation of the mitochondrial chaperone cyclophilin D [108]. This effect also provides a link between GSK-3*β* and oxidative stress. Studies have also focused on the link between PP2A and oxidative stress. A recent report shows that rat cortical neurons treated with okadaic acid inhibit PP2A activity, resulting in an abnormal increase in mitochondrial ROS and mitochondrial fission [109]. Other findings reveal that ROS inhibits PP2A and PP5, leading to the activation of JNK and Erk1/2 pathways and subsequently caspase-dependent and caspase-independent apoptosis of neuronal cells [110]. Despite these studies, the relationship of GSK3 and PP2A with oxidative stress remains to be further investigated.

#### 3.3.3. Antioxidants and the Tau Protein

Several epidemiological studies have indicated a link between antioxidant intake and reduced incidence of dementia (particularly AD) and cognitive decline in elderly populations [111–113]. In recent years, antioxidant therapy has received considerable attention as a promising approach for slowing the progression of AD. Research has focused on endogenous antioxidants (e.g., vitamins, coenzyme Q10, and melatonin) and the intake of dietary antioxidants, such as phenolic compounds that are flavonoids or nonflavonoids [114, 115]. This increased interest has thus strengthened the hypothesis that oxidative damage may be responsible for the cognitive and functional decline in AD patients. Melatonin is a free radical scavenger that blocks tau hyperphosphorylation and microtubule disorganization under in vivo and in vitro conditions [116–118]. It also decreases the activity of GSK-3*β* [119]. Moreover, melatonin may be a potentially useful agent in the prevention and treatment of AD [120]. Other antioxidants, such as vitamins E and C [121, 122], gossypin [123], curcumin [124–127], beta-carotene [128], and* Ginkgo biloba* [129, 130], are also reported to have a protective effect against neurotoxicity. In addition, an association also exists between beta-carotene and tau in AD patients [128]. Demethoxycurcumin has been shown to inhibit the phosphorylation of both tau pS(262) and pS(396) in murine neuroblastoma N2A cells [125]. Curcumin also reduces soluble tau and elevated heat shock proteins involved in tau clearance [126]. These results have therefore led to further investigations of this compound as an antioxidant therapy strategy for AD. Other experiments have shown that the active component of* Ginkgo biloba*, ginkgolide A, inhibits GSK3*β* and suppressed the phosphorylation level of tau [129].

## 4. Autophagy in AD

### 4.1. The Autophagic Pathway

Autophagy is an essential lysosomal degradation pathway that turns over cytoplasmic constituents, including misfolded or aggregated proteins and damaged organelles, to facilitate the maintenance of cellular homeostasis [13, 131–134]. Autophagy is usually activated during nutrient deprivation and stress to enhance cellular survival, and its constitutive activity is recognized to control neuronal survival [14, 132, 135, 136]. Autophagic dysfunction has been reported to contribute to AD [20, 64, 137, 138].

Autophagy includes macroautophagy, chaperone-mediated autophagy, and microautophagy [13, 132, 134]. The most familiar of these types is macroautophagy, which is a process of cellular self-cannibalism in which portions of the cytoplasm are sequestered within double- or multimembraned vesicles (autophagosomes) and then delivered to lysosomes for bulk degradation [139]. Autophagy is induced by two pathways in macroautophagy-mammalian target of rapamycin- (mTOR-) dependent and mTOR-independent signaling pathways [140]. mTOR is an important convergence point in the cell signaling pathway. mTOR kinase activity is modulated in response to various stimuli, such as trophic factors, mitogens, hormones, amino acids, cell energy status, and cellular stress [135, 136]. Rapamycin, as mTOR inhibitor, is a very important tool for autophagy [140, 141]. mTOR complex (mTORC) 1 is involved in autophagy and is the master regulator of cell growth enhancing the cellular biomass by upregulating protein translation [142]. For cells to control cellular homoeostasis during growth, a close signaling interplay occurs between mTORC1 and two other protein kinases, AMP-activated protein kinase (AMPK) [143] and Unc51-like kinase (ULK1) [144]. Autophagy is inhibited by cytosolic p53 via the direct inhibition of AMPK [145]. mTORC1 controls autophagy by directly interacting with the Ulk1-focal adhesion kinase family-interacting protein of 200 kDa (Atg13-FIP200) complex [146]. Several mTOR-independent signals affect the autophagy pathway. When the level of free inositol and myoinositol-1,4,5-trisphosphate IP3 decreases, autophagy is reduced [147]. Furthermore, lower levels of Bcl-2 lead to the release of more Beclin-1, thus forming the Beclin-1-PI3KCIII complex to activate autophagy via the PI3K-AKT-mTOR pathway [148].

### 4.2. Autophagic Dysfunction in AD Pathology

A growing body of evidence suggests a link between AD and autophagy [16, 17, 19, 20, 22]. Therefore, the pathological functions of autophagy may be a critical mediator of neurotoxicity [149]. Autophagy develops in AD brains because of the ineffective degradation of autophagosomes, which is controlled by many kinds of autophagy-related genes (Atg), including Atg1–Atg35. Atg8 (mammalian homolog is LC3) is an autophagosomal membrane protein and a marker of autophagosome formation [150]. Beclin-1 (the mammalian ortholog of yeast Atg6) plays a pivotal role in autophagy [151]. In an in vitro study of the pathogenesis of AD, Atg8/LC3 colocalizes with APP and LC3-positive autophagosomes are present [152]. Beclin-1 knockdown increases APP, APP-like proteins, APP-C-terminal fragments, and A*β* [153]. Atg5, Atg12, and LC3 are also associated with plaque, tangle pathologies, and neuronal death in AD [154]. Generally, autophagic vacuoles (AVs) are rare in the normal brain but are increased in brains of AD patients. In the early stages of AD, the expression of lysosome-related component is significantly increased prior to the formation of plaques and NFTs, and autophagy is also induced at this stage; thus its activity is independent of extracellular A*β* deposition and NFT formation [155]. In the late stage of AD, AVs continue to accumulate in large numbers in dystrophic neurites. There are several causes for the dysfunction of autophagy in late-stage AD, including the enhanced processing of APP and A*β* degradation [156] and the toxic effect of high levels of intracellular A*β* on lysosomal function [157]. Inhibition of the AV-lysosome fusion is caused by impaired microtubule-associated retrograde transport, which in turn leads to increased accumulation of AV in dystrophic neurites [134]. Lysosomal enzyme dysfunction may be associated with the accumulation of AVs [158]. Autophagy plays an important role in the degradation of impaired mitochondria in AD [158, 159]. Dysfunction of the autophagy-lysosome system causes insufficient degradation of mitochondria [160]. Conversely, mitochondrial dysfunction may also impair this pathway [161].

### 4.3. Autophagy and the Tau Protein

#### 4.3.1. Tau Protein Degradation via Autophagy

A variety of forms of tau proteins have been shown to be degraded by the ubiquitin-proteasome system (UPS) and autophagy-lysosome system. UPS may play an important role in the primary clearance of pathological tau. However, the importance of autophagy-mediated tau degradation, particularly at the late stage of NFT formation, is becoming more recognized. The autophagy-lysosomal system has the capacity to engulf protein aggregates and keep tau levels at a low level [162]. Macroautophagy is believed to be an evolutionarily conserved mechanism for intracellular degradation of proteins, such as A*β* and tau. mTOR in negatively regulating autophagy is an important convergence point in cell signaling. Increasing mTOR signaling and PI3K/AKT/mTOR pathway facilitates tau pathology, but reducing this signaling ameliorates tau pathology [11, 20, 163]. Rapamycin has been reported to decrease tau phosphorylation at Ser214 in vitro and reduce tau tangles and insoluble tau in vivo [164, 165]. In a tetracycline-inducible model [tauDeltaC (tauΔC)], tau is abnormally truncated at Asp^421^ and is cleared predominantly by macroautophagy and degraded significantly faster than full-length tau [166]. Autophagy activation suppresses tau aggregation and eliminates cytotoxicity [163]. Moreover, trehalose (an enhancer of autophagy) directly inhibits tau aggregation in primary neurons [167]. Under in vitro conditions, the accumulation of tau species is increased with the autophagic inhibitor, 3-methyladenine, and decreased with trehalose [168]. Overall, these results suggest that tau degradation involves autophagy, and this activity is beneficial for neurons to prevent the accumulation of protein aggregates.

#### 4.3.2. Tau Protein Hyperphosphorylation Leads to Autophagic Dysfunction

The physiological function of tau protein is well known to be associated with microtubule binding and assembly. Autophagosome transport mainly depends on the movement along microtubules in the autophagic pathway. However, the link between tau hyperphosphorylation and autophagic dysfunction is still under debate. Frontotemporal dementia and parkinsonism linked to chromosome 17- (FTDP-17-) mediated tau mutations can disrupt lysosomal function in transgenic mice expressing human tau with four tubulin-binding repeats (increased by FTDP-17 splice donor mutations) and three FTDP-17 missense mutations: G272V, P301L, and R406W [169]. In Tg mice expressing mutant human (P301L) tau, axonal spheroids have been shown to contain tau-immunoreactive filaments and AVs [170]. A recent study has revealed that PP2A upregulation stimulates neuronal autophagy, thus providing a link between PP2A downregulation, autophagy disruption, and protein aggregation [171]. Furthermore, autophagosomes have been shown to be increased in rat neurons treated with okadaic acid [172]. Altogether, tau is known to regulate the stability of microtubules, and tau hyperphosphorylation may result in the destabilization of neuronal microtubules, thus affecting the placement and function of mitochondria and lysosomes. Therefore, tau hyperphosphorylation is likely to play a critical role in the process of autophagic dysfunction.

#### 4.3.3. Autophagic Dysfunction Induces Tau Protein Aggregation and Neurodegeneration

The autophagy-lysosome system is well recognized to play an important role in the clearance of abnormally modified proteins in cells. Several studies have shown that dysfunction of the autophagy-lysosome system contributes to the formation of tau oligomers and insoluble aggregates [22, 173, 174]. Abnormal lysosomal proteases are also found in brains of AD patients [173, 174]. Both phosphorylated tau and GSK3*β* significantly accumulate in Atg7 conditional knockout brains, although NFTs are absent [20]. The hyperphosphorylation of tau and NFT formation result in the disruption of the neuronal skeleton, thereby contributing to neuronal dysfunction, cell death, and eventually the symptoms of AD. Genetic reduction of mammalian target of rapamycin led to an increase in autophagy induction and ameliorates Alzheimer's disease-like cognitive and pathological deficits [22]. Induction autophagy adaptor protein NDP52 may reduce tau protein phosphorylation in neurons [20]. Therefore, the autophagy-lysosome system plays a crucial role in the clearance of tau, and its accumulation may be due to autophagic dysfunction in cells.

## 5. Conclusion

Oxidative stress is reported to be one of the earliest events in AD and can induce tau hyperphosphorylation, which destabilizes microtubules by decreasing the binding affinity of tau, thereby resulting in the formation of NFTs, which are a major pathological hallmark of AD. A*β* and other risk factors play the crucial role in neuronal oxidative stress. A*β*-mediated mitochondrial oxidative stress causes hyperphosphorylation of tau in AD brains, as well as mitochondrial dysfunction. Tau hyperphosphorylation may be the necessary point in neural dysfunction and death. Hyperphosphorylation, oligomerization, fibrillization, and propagation of tau pathology have been proposed as the pathogenesis of tau-mediated neurodegeneration. In addition to oxidative stress, tau protein phosphorylation is also regulated by protein kinase and phosphatase. It indicates the roles of mitochondria and protein phosphatase on oxidative stress and tau protein hyperphosphorylation. Meanwhile it strengthens the hypothesis that oxidative damage is responsible for the cognitive and functional decline in AD patients.

Dysfunctional tau protein is degraded via autophagy-lysosomal pathway. Autophagy is an essential lysosomal degradation process that turns over cytoplasmic constituents, including misfolded or aggregated proteins and damaged organelles, to facilitate the maintenance of cellular homeostasis. Tau hyperphosphorylation is likely to play a critical role in the process of autophagic dysfunction, and dysfunction of the autophagy-lysosome system may also promote the tau aggregation. Altogether, tau is known to regulate the stability of microtubules, and tau hyperphosphorylation may result in the destabilization of neuronal microtubules, thus affecting the function of mitochondria and lysosomes.

**Figure 1:**
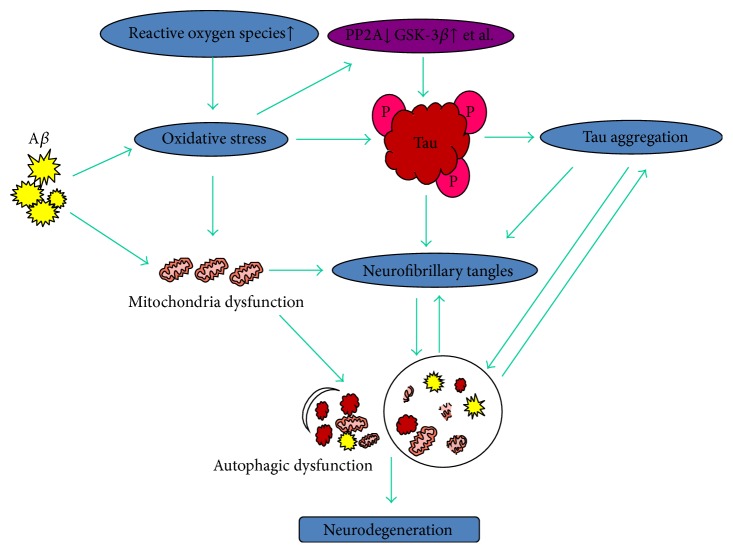
Tau protein NFTs formation and autophagic dysfunction in Alzheimer's disease. A*β* oligomers and ROS production intrigue oxidative stress and mitochondria dysfunction, in which induce tau protein hyperphosphorylation and neurofibrillary tangles formation with protein phosphatase and kinases imbalance. These events converge to autophagic dysfunction and tau protein aggregation to lead to neurodegeneration and cell death in AD.

These events initiate a series of cascades to induce neurodegeneration and cell death in AD. A*β* oligomers and ROS production intrigue oxidative stress and mitochondria dysfunction, in which they induce tau protein hyperphosphorylation and neurofibrillary tangles formation with protein phosphatase and kinases imbalance. These events converge to autophagic dysfunction and tau protein aggregation to lead to neurodegeneration and cell death in AD (Figure 1). However, the relationships between oxidative stress, tau hyperphosphorylation, and autophagic dysfunction and accurate mechanisms on neurodegeneration, especially mitochondria and protein phosphatase in AD, still require further research.
